# The effect of virtual reality technology on anti-fall ability and bone mineral density of the elderly with osteoporosis in an elderly care institution

**DOI:** 10.1186/s40001-023-01165-9

**Published:** 2023-06-29

**Authors:** Rui Zhao, Xiangdi Zhao, Jianzhong Guan, Changchun Zhang, Kun Zhu

**Affiliations:** 1 Department of General Medicine, Bengbu Medical College, Bengbu, China; 2 Department of Orthopaedics, The First Affiliated Hospital of Bengbu Medical College, No. 287, Changhuai Road, Bengbu, 233000 Anhui China; 3 School of Pharmacy, Bengbu Medical College, Bengbu, China; 4 Department of Orthopaedic Surgery, Nanjing First Hospital, Nanjing Medical University, Nanjing, China

**Keywords:** Virtual reality, Elderly care institutions, Osteoporosis, Falls, Bone mineral density

## Abstract

**Objective:**

To explore the impact of virtual reality (VR) training on anti-fall ability and bone mineral density (BMD) among elderly patients admitted to a healthcare institution.

**Methods:**

People (aged 50) with osteoporosis in an elderly care institution in Anhui Province June 2020 to October 2021 were selected and randomly divided into VR group (*n* = 25) and control group (*n* = 25). In VR group, the virtual reality rehabilitation training system was used for training, while control group was treated with traditional fall prevention exercise intervention. The changes of Berg Balance Scale (BBS), timed up and go test (TUGT), functional gait assessment (FGA), bone mineral density (BMD) and falls during 12 months of training were compared between the two groups.

**Results:**

BBS and FGA were positively correlated with BMD of the lumbar vertebrae and femoral neck, and TUGT was negatively correlated with BMD of the lumbar vertebrae and femoral neck. After 12 months of training, the BBS score, TUGT evaluation and FGA evaluation of the two groups were significantly improved compared with those prior to training (*P* < 0.05). However, there was no significant difference in the lumbar spine and femoral neck BMD between the two groups 6 months after the intervention. The femoral neck and lumbar spine BMD of the VR group improved, and it was significantly higher than that of the control group 12 months after the intervention. Nevertheless, there was no significant difference in terms of the incidence of adverse events between the two groups.

**Conclusion:**

VR training can improve anti-fall ability and increase femoral neck and lumbar spine BMD and can effectively prevent and reduce the risk of injury among elderly people with osteoporosis.

## Introduction

With the increasing aging population worldwide, osteoporosis has become a major social issue, and most patients with osteoporosis develop different complications [1]. Moreover, osteoporotic fractures caused by falls often lead to serious complications among people aged over 65 years [2, 3]. Lack of regular exercise and a natural decline in physiological function are associated with osteoporosis, which is defined as decreased muscle strength and anti-fall ability, and a high risk of falling [4]. Mechanical stimulation of skeletal muscles is indispensable for bone homeostasis, and lack of mechanical stimulation can reduce bone formation, leading to osteoporosis and a high risk of fractures [5].

Currently, pharmacological intervention is the main option for treating osteoporosis among elderly individuals [6]. The VR technology has been successfully applied to different fields of medical and health care, such as sports rehabilitation and psychotherapy, for elderly individuals [7, 8]. Therefore, the implementation of innovative and effective strategies to improve the anti-fall ability and the bone mineral density (BMD) of elderly people with osteoporosis is a public health challenge that should be addressed urgently.

In elderly healthcare institutions, people without family support are more likely to experience accidental falls, thereby causing fractures. With improvement in science and technology by leaps and bounds, the development of novel technologies for smart elderly care has become a novel trend in response to the global challenges of aging [9]. The current study aimed to evaluate the effects of VR technology and calcium supplementation on anti-fall ability and BMD among elderly individuals with osteoporosis admitted to a healthcare institution. We hypothesized that exercise applied by VR techniques plus oral calcium supplementation can improve Berg Balance Scale (BBS), Timed Up and Go Test (TUGT), and Functional Gait Assessment (FGA) scores and increase lumbar spine and femoral neck BMD, thereby preventing and reducing the risk of injury among elderly individuals.

## Materials and methods

This research enrolled 50 patients with osteoporosis admitted to an elderly healthcare institution in Anhui Province, China, from June 2020 to October 2021. Moreover, it was designed and conducted by the Department of General Medicine, Bengbu Medical College. All participants provided a written informed consent form, and the study was approved by the Ethics Committee of The First Affiliated Hospital of Bengbu Medical College and was performed in accordance with the principles of the Declaration of Helsinki.

### Inclusion and exclusion criteria

The inclusion criteria were as follows: (1) individuals aged ≥ 65 years, (2) those who can walk independently or with assistive devices, (3) those with a lower limb muscle strength of ≥ level III and sitting and standing balance of ≥ level II [10], (4) those who underwent routine assessment of the lumbar spine and femoral neck BMD (T-score <  − 2.5 SD), and (5) those who have normal cognitive ability and can understand and cooperate with the training. The exclusion criteria were as follows: (1) individuals with severely impaired vision or hearing, (2) those with neurological diseases affecting balance ability, (3) those with lower limb injury or a history of surgery, (4) those with severe cardiopulmonary and vascular diseases, (5) those with different acute-phase or acute-onset chronic diseases, and (6) those with any type of waist or hip fracture.

### Research participants

This was a randomized two-arm, subject-blinded, controlled, single-center clinical trial. The participants were randomly divided into the control (*n* = 25) and VR (*n* = 25) groups using the random number table method. Among them, 11 presented with high blood pressure, 10 with diabetes, 8 with respiratory disease, 3 with heart disease, and 2 with digestive system disease (Table 1).

**Table 1:** Comparison of the general condition of the two groups

Items	VR group	Control group	t/χ^2^	*P*-value
Cases	25	25		
Age (years)	72.16 ± 3.64	73.36 ± 3.25	1.230	0.225
Sex (M/F)	13/12	8/17	2.053	0.152
BMI	26.37 ± 6.49	27.10 ± 3.67	0.490	0.626
Lumbar BMD	0.745 ± 0.034	0.746 ± 0.045	0.035	0.972
T-score	− 2.82 ± 0.41	− 2.79 ± 0.49	0.251	0.803
Femoral neck BMD	0.592 ± 0.029	0.594 ± 0.027	0.255	0.800
T-score	− 2.23 ± 0.61	− 2.26 ± 0.58	0.191	0.849
Fracture history (Y/N)	24/1	22/3	0.272	0.602
Osteoarthritis history(Y/N)	15/10	19/6	0.827	0.363
Application of auxiliary devices (Y/N)	11/14	13/12	0.321	0.571
Current smoking (Y/N)	6/19	7/18	0.104	0.747
Alcohol consumption (Y/N)	3/22	5/20	0.149	0.700
Physical activity				
Low	10	12	1.070	0.586
Moderate	8	9		
High	7	4		
Diseases				
High blood pressure	6	5		
Diabetes	3	7		
Respiratory disease	5	3	0.368	0.544
Heart disease	2	1		
Digestive system disease	2	0		

### Intervention methods

Subjects in both groups received calcium routinely as the basic anti-osteoporosis treatment (vitamin D calcium chewable tablets with 100 IU of Vit D, 200 IU/day).

In control group, subjects performed routine fall prevention exercises under the leadership of an instructor, including medium-intensity aerobic gymnastics and apparatus exercises, with 50 min each time, 3 times per week, lasting for 12 months. The goal is to achieve core muscle training, lower extremity muscle strength, balance and gait function training.

In VR group, a VR rehabilitation training system (Nanjing Moxun Company, Motion2.0) was used for training. The subjects wore eye masks and were received 3 kinds of VR sports games: skiing, diving, and running. The training regimen was 3 times per week, 50 min each time (including 5-min of warm-up exercise, 40-min of VR sports game training and 5-min of organized exercise), with total training period of 12 months.

### Data collection

The International Physical Activity Questionnaire-Short Form (IPAQ-SF) was used to measure Physical activity levels before training [11].

All subjects were tested for anti-fall parameters and BMD before training, 6 months and 12 months post training.

Anti-fall parameters include: Berg balance scale (BBS); Timed up and go test (TUGT); Functional gait assessment (FGA).

Bone mineral density (BMD) measurement: The BMD of patient’s femoral neck and lumbar vertebrae were measured using a dual-energy X-ray absorptiometry (Hologic Discovery W series). The total spine and Femoral Neck least significant change (LSC) were 2.6% and 4.1%, respectively. And all operators were professionally trained and obtained the training certificate issued by The International Society for Clinical Densitometry.

During the training period, all subjects were observed to count the number of falls and the fracture situation after the fall were tracked.

### Statistical analysis

SPSS 22.0 was used for data entry and statistical processing, and GraphPad Prism 8.0 was used for graphing. Percentage changes of data were expressed as mean ± standard deviation ($\overline{\mathrm{x} }$ ± SD). The independent t-test was used to assess differences in percentage changes between the groups during follow-up and differences in pre–post changes between the groups. Correlation analysis adopts Pearson correlation analysis. The *P*-value of < 0.05 was considered significant.

## Results

### Characteristics of the participants

This study screened 72 participants. Among them, 9 did not meet the inclusion criteria, and 12 refused to participate. Finally, 51 participants were enrolled. One patient in the VR group did not undergo the training and dropped out of the study. The attendance rate of the VR group was 96.15%. All participants complied with the training program. Results showed no significant differences between the two groups in terms of age, sex, body mass index, application of auxiliary devices, incidence of comorbidities, and current smoking and alcohol consumption (*P* > 0.05) (Table 1).

### Correlation analysis between BMD of lumbar vertebrae and femoral neck and BBS score, TUGT score, and FGA score when all subjects were enrolled

**Fig. 1:**
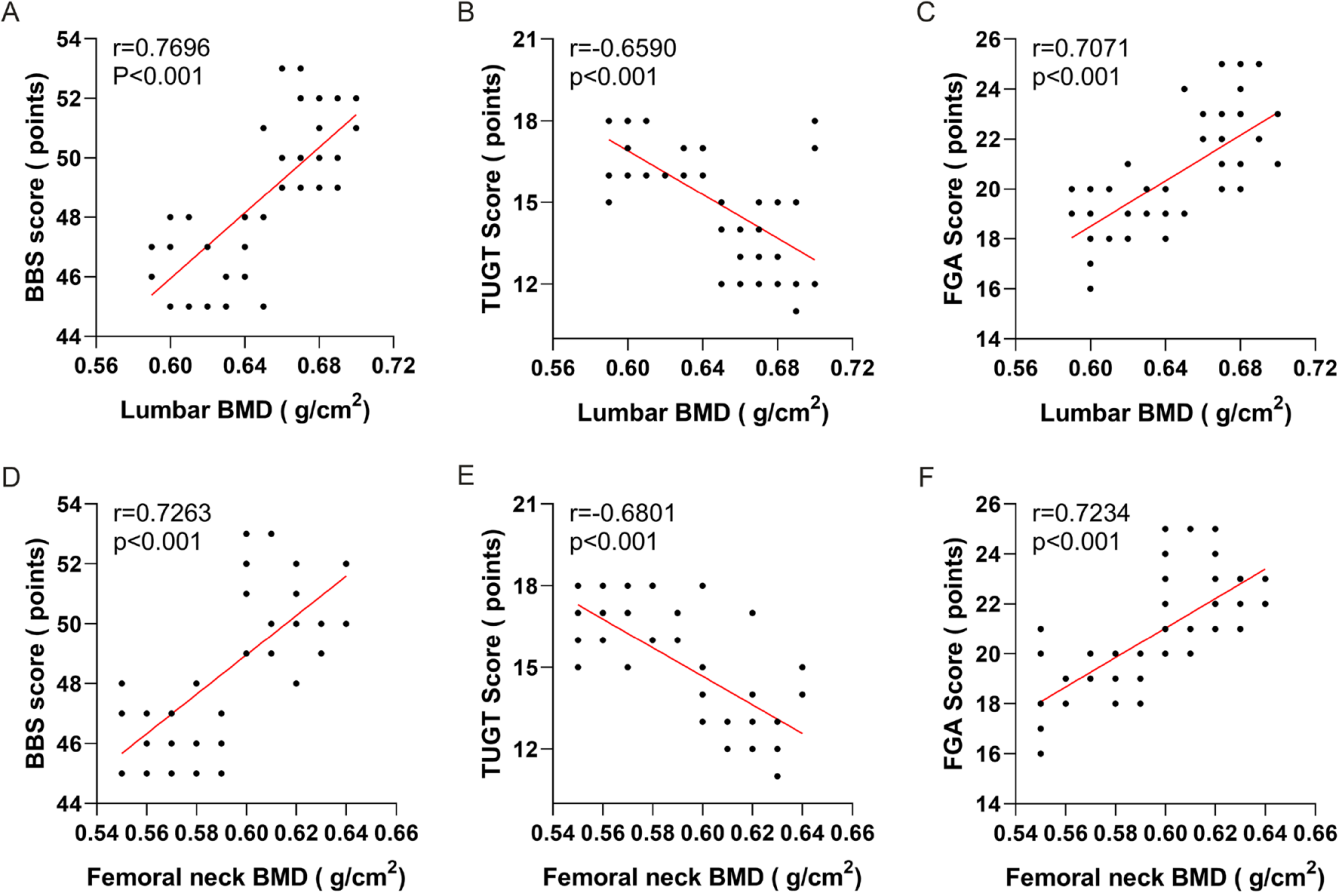
**A** BBS score was positively correlated with lumbar BMD; **B** TUGT score was negatively correlated with lumbar BMD; **C** FGA score was positively correlated with lumbar BMD; **D** BBS score was positively correlated with femoral neck BMD; **E** TUGT score was negatively correlated with femoral neck BMD; **F** FGA score was positively correlated with femoral neck BMD

When all subjects were enrolled, BBS score and FGA score were positively correlated with lumbar BMD, and TUGT score was negatively correlated with lumbar BMD; when all patients were enrolled, BBS score and FGA score were positively correlated with femoral neck density, while TUGT score is negatively correlated with femoral neck density. (Fig. 1).

### Anti-fall indicators of the VR and control groups before and during the intervention

**Fig. 2:**
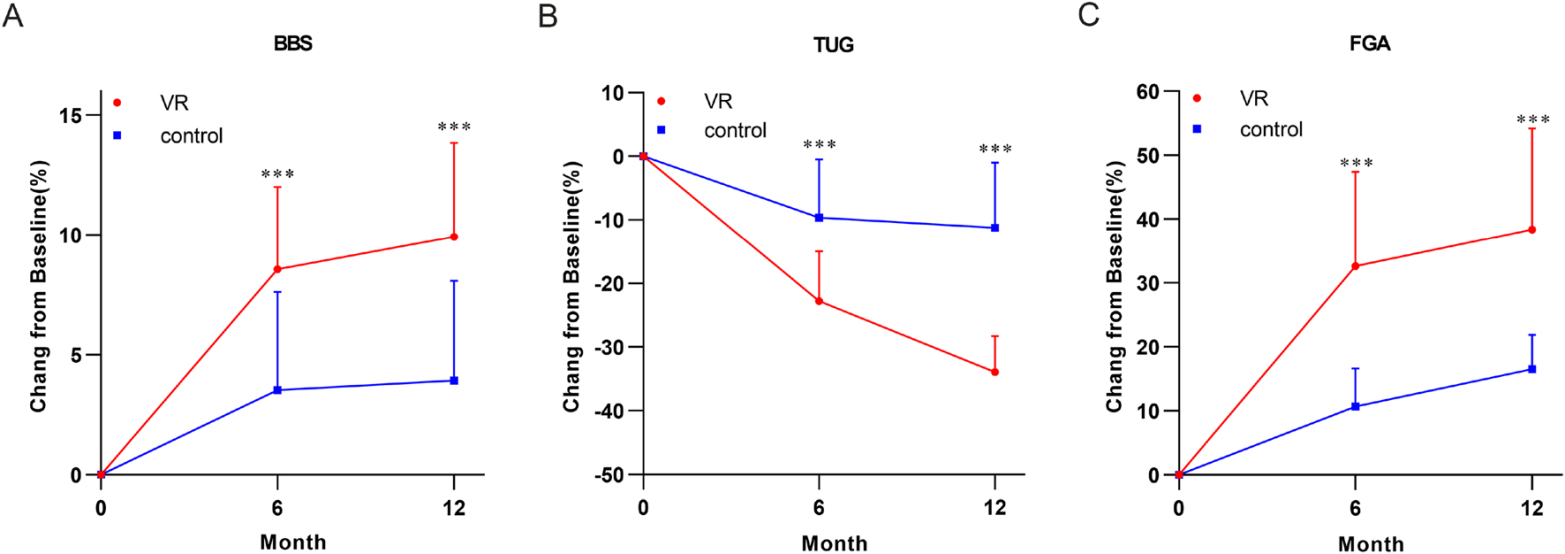
Percent change of BBS, TUG and FGA after intervention. Results are shown for the percent changes of BBS (**A**), TUG (**B**), FGA (**C**). Data are presented as the mean ± SD. ***P < 0.001

There were no significant differences in terms of BBS, TUG, and FGA scores between the control and VR groups before the intervention. The BBS and FGA scores of the VR group increased significantly 6 months after the intervention compared with those of the control group (8.6%, 95% confidence interval [CI] 7.1–10.0, *P* < 0.001 and 32.6%, 95% CI 26.5–38.7, *P* < 0.001 vs 3.5%, 95% CI 1.8–5.2 and 10.7%, 95% CI 8.2–13.1). The TUG of the VR group decreased significantly compared with that of the control group (22.8%, 95% CI 19.6–26.0, *P* < 0.001 vs 9.6%, 95% CI 5.9–13.4). The BBS and FGA scores of the VR group increased significantly 12 months after the intervention compared with those of the control group (9.9%, 95% CI 8.3–11.5, *P* < 0.001 and 38.4%, 95% CI 31.8–44.9, *P* < 0.001 vs 3.9% CI 2.2–5.6 and 16.5%, 95% CI 14.3–18.7) The TUG score of the VR group decreased significantly compared with that of the control group (33.9%, 95% CI 31.6–36.3, *P* < 0.001 vs 11.2%, 95% CI 7.0–15.5) (Fig. 2).

### BMD of the two groups before and during the intervention

**Fig. 3:**
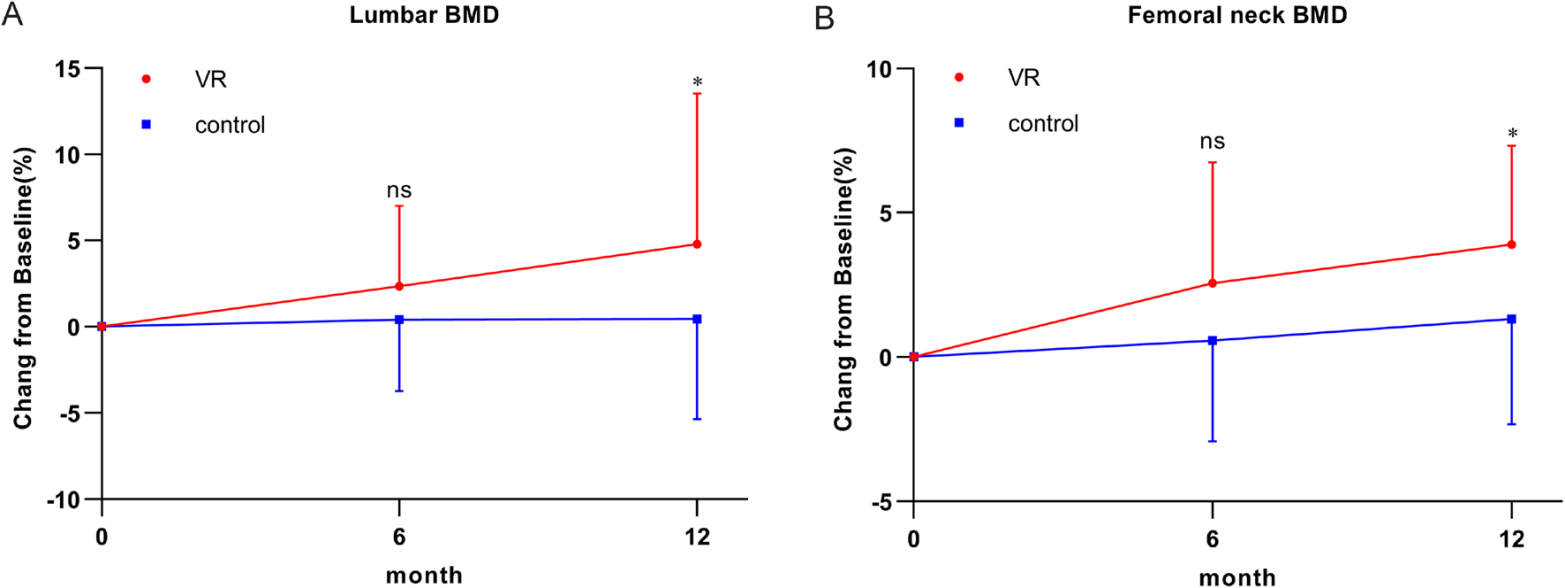
Percent change of BMD at lumbar spine and femoral neck after intervention. Results are shown for the percent changes of BMD at lumbar spine (**A**), femoral neck (**B**). Data are presented as the mean ± SD. *P < 0.05, ns: no significance

There were no significant differences in terms of the lumbar spine and femoral neck BMD between the control and VR groups before the intervention. Moreover, the lumbar spine and femoral neck BMD did not significantly differ between the VR and control groups (2.3% and 2.6% vs 0.4% and 0.6%). The lumbar spine and femoral neck BMD of the VR group increased significantly 12 months after the intervention compared with that of the control group (4.8%, 95% CI 1.2–8.4, *P* = 0.045 and 3.9%, 95% CI 2.5–5.3, *P* = 0.013 vs 0.5% and 1.3%) (Fig. 3).

### Observation for falls during 12-month follow-up visits

After training, within a follow-up period of 12 months, two subjects in control group had a fall, one of whom experienced a distal radius fracture after falling, while in VR group, one subject experienced a fall but without fracture occurred.

## Discussion

The current study aimed to evaluate the effect of exercise applied by VR techniques on improving anti-fall ability and lumbar spine and femoral neck BMD among elderly individuals admitted to a healthcare institution. Results showed that exercise applied by VR techniques was more effective in improving the anti-fall ability and BMD of elderly patients than traditional training. To the best of our knowledge, the number of similar studies was extremely limited. This research innovatively applied exercise applied by VR techniques in the training of elderly individuals admitted to a healthcare institution. Moreover, changes in anti-fall ability and BMD were evaluated, and a novel approach for the treatment of elderly individuals with osteoporosis admitted to a healthcare institution was introduced.

Studies have indicated that [12, 13] an increase in physical activity can enhance the metabolism of the elderly and increase the metabolic rate of bone, subsequently promoting the proliferation of osteoblasts and reducing the number of osteoclasts, ultimately enhancing the function of bone production. And BMD has also increased accordingly. In addition, skeletal muscle-derived exosomes can reverse bone loss [14]. During exercises training, skeletal muscle contraction released a large number of exosomes, which improved osteoporosis symptoms [15]. In this study, the BBS score, TUGT score, FGA score and lumbar vertebrae BMD and femoral neck BMD were analyzed by Pearson correlation analysis of all subjects prior to enrollment. The BMD of lumbar vertebrae and femoral neck were positively correlated with both BBS and FGA score, while negatively correlated with TUGT evaluation, indicating that osteoporotic patients with good balance ability and lower risk of falling have higher BMD in lumbar vertebrae and femoral neck. This also suggested that improvement of balance ability after exercise had a better effect on prevention of osteoporosis in the elderly.

Elderly individuals presented with decreased athletic ability, including coordination, balance, muscle strength, and speed [16, 17]. The BBS focuses on balance and mobility tests of subjects. TUGT mainly calculates the time of standing and walking, and FGA can determine whether there is a balance disorder in the subjects. The VR technology sports training had a positive effect on athletic ability. That is, it improved the athletic ability of elderly individuals by promoting motor skills, sensorimotor learning, and cortical plasticity [18, 19]. Previous studies have used exercise applied by VR techniques in community and home-based settings. Results showed that exercise applied by VR techniques increased the sports training potential and significantly improved the static and dynamic balance of elderly individuals. In addition, VR fitness game exercises continuously stimulated elderly individuals and maintained their motivation to repeatedly perform different types of exercises. This increases the amount of training and improves balance ability, which is significantly important in preventing falls [20–23]. The current study showed no significant difference between the two groups in terms of BBS, TUGT, and FGA scores before training. However, the BBS, TUGT, and FGA scores significantly differed after the 6- and 12-month training. Hence, exercise applied by VR techniques can effectively improve balance ability, gait coordination, and lower limb muscle strength among elderly individuals, thereby improving anti-fall abilities. After the 6- and 12-month training, the VR group had better BBS, TUGT, and FGA scores than the control group. Hence, compared with traditional sports training, VR sports training was more effective in preventing falls among elderly individuals admitted to a healthcare institution.

With aging, bone fragility increases, and BMD decreases. These factors mainly cause fractures. Previous studies have found that adverse factors including decreased exercise capacity and muscle strength affect BMD among elderly individuals [23, 24]. Moreover, the establishment of a scientific aerobic exercise plan and the regular monitoring of exercise capacity and exercise volume can increase bone mineralization and bone calcium absorption and promote cell activity [12, 25]. Previous studies have shown that increased physical activity can enhance metabolism and increase the bone metabolic rate in elderly individuals. These mechanisms subsequently promote the proliferation of osteoblasts and reduce the number of osteoclasts, thereby subsequently enhancing the function of bone production and increasing BMD [15, 26]. Furthermore, skeletal muscle-derived exosomes can reverse bone loss [15, 27]. During exercise training, skeletal muscle contraction releases a high level of exosomes, which improves osteoporosis symptoms [15, 28]. In the current study, the percentage changes in the lumbar spine and femoral neck BMD of the VR and control groups before and during training were evaluated and compared. Results showed that the lumbar spine and femoral neck BMD of the VR group increased significantly after the 12-month training. However, the lumbar spine and femoral neck BMD of the control group did not increase significantly after the 6-month training.

VR intervention can cause vertigo symptoms, which can be relieved by removing the headset. This type of vertigo is attributed to the conflict between what your eyes see and the information felt by the vestibular system of the inner ear [29]. Elderly participants who had falls may experience fear of falling, post-fall anxiety syndrome, depression, and reduced activity. Injuries sustained after falls have an extremely serious negative impact on health [22, 25]. Some studies have found that exercise applied by VR techniques can significantly reduce the number of falls compared with traditional balance training [30]. In the current study, elderly patients were followed up for 12 months. Five people in the VR group experienced vertigo. Among them, one did not continue with the training after adjustment for 1 week and withdrew from the study. However, none of the patients in the control group dropped out of the study. One patient in the VR group and two in the control group experienced falling. One patient in the control group sustained a fracture after falling. However, there was no significant difference in the incidence of adverse events between the two groups.

The current study had several limitations. That is, the follow-up period was short. Nonetheless, improvement in BBS, TUGT, and FGA scores was consistent with that of previous studies. Hence, there was no significant difference in the incidence of adverse events between the VR and control groups. Nevertheless, the current study also had several strengths. That is, the effect of VR training on BMD among elderly individuals was investigated, and no similar study has been conducted thus far.

## Conclusion

In conclusion, the use of VR technology in sports training can increase BMD and improve anti-fall ability among elderly patients with osteoporosis admitted to healthcare institutions. Moreover, it can be beneficial for the promotion of smart elderly care during the global health crisis.

## Data Availability

All data generated or analyzed during this study are included in this manuscript.
